# Severe Hypokalemia Causing Ventricular Tachycardia: A Case Report

**DOI:** 10.7759/cureus.34043

**Published:** 2023-01-21

**Authors:** Katharine Pula, Kedar N Patel, Robert P Briggs, Kevin R Weaver

**Affiliations:** 1 Department of Emergency and Hospital Medicine, Lehigh Valley Health Network/University of South Florida Morsani College of Medicine, Allentown, USA

**Keywords:** torsade de pointes, hypokalemia, wide-complex tachycardia, neurological deficits, electrolyte abnormality, hyperosmolar hyperglycemic syndrome, ventricular tachycardia

## Abstract

Hypokalemia and hyperosmolar hyperglycemic syndrome (HHS) are two reversible but potentially fatal disorders that are important to identify and treat urgently. A 43-year-old patient presented to the ED with altered mental status and slurred speech, difficulty communicating, left-sided facial droop, and stool incontinence according to emergency medical services. This was preceded by 1.5 weeks of nausea, vomiting, polydipsia, and weight loss. On presentation, the patient was found tachycardic and tachypneic, with uncertain neurological deficits on physical exam, hyperglycemia, and electrocardiogram (EKG) abnormalities. Lab data were consistent with hyperosmolar hyperglycemic nonketotic coma.

This case provides two important clinical scenarios in which cardiac EKG abnormalities and focal neurological deficits are the product of hyperosmolality and electrolyte abnormalities. Hypokalemia with EKG abnormalities consistent with a potential ischemic disease can progress into wide complex tachycardia and ventricular fibrillation. Hyperosmolar hyperglycemia may manifest with focal neurological deficits and without the classical presentation of a coma. Careful consideration of EKG and lab values in the context of clinical presentation may provide clues to resolvable etiologies. We report a case of a patient who presented to the ED with hypokalemia and HHS, both reversible but potentially fatal disorders that are important to identify and urgently treat.

## Introduction

Hypokalemia is a common electrolyte abnormality encountered in clinical practice. Serum potassium levels indicate the severity of hypokalemia: mild is 3-3.4 mmol/L, moderate is 2.5-3 mmol/L, and severe is less than 2.5 mmol/L [1]. The etiology of hypokalemia comes from three main mechanisms: decreased intake, transcellular shifts, and potassium loss (skin, GI, or renal). Clinical manifestations of hypokalemia do not present until levels are under 3 mmol/L and include cardiac arrhythmias, muscle weakness, constipation, and fatigue [1]. Electrocardiogram (EKG) manifestations of hypokalemia are initiated by decreased T-wave amplitude, followed by ST-interval depression, T-wave inversions, PR-interval prolongation, and U waves [2]. U-wave abnormalities are present in almost 80% of patients with serum potassium levels less than 2.7 mmol/L [3]. The U-wave is best visualized in the mid-precordial leads (leads V1-V4) and with severe hypokalemia may mask the preceding T-wave or following P-waves [4,5]. Hypokalemia leads to direct electrophysiological effects of early afterdepolarization and prolonged action potential duration, which may culminate into dysrhythmias such as ventricular tachycardia (VT) or fibrillation and Torsades de Pointes (TdP) [4]. In patients with EKG features of hypokalemia, urgent treatment is recommended with potassium replacement and magnesium sulfate injection to prevent early afterdepolarizations [6].

Hyperosmolar hyperglycemic syndrome (HHS) is a particularly devastating condition that carries a high mortality risk (up to 20%) in the adult population [7]. We report a case of a patient who presented to the emergency department (ED) with hypokalemia and HHS, both reversible but potentially fatal disorders that are important to identify and treat urgently.

## Case presentation

A 43-year-old male presented to the ED with altered mental status. Per emergency medical services (EMS), the patient had slurred speech, difficulty communicating, and left-sided facial droop with stool incontinence. EMS also reported four empty haloperidol bottles with unidentified pill contents were found near the patient. The patient’s family reported he had nausea, vomiting, polydipsia, and weight loss for the past 1.5 weeks. The family also reported the patient had a history of alcohol use. His past medical history was only remarkable for bipolar disorder, for which he was not prescribed any selective serotonin reuptake inhibitors (SSRIs). The patient was prescribed 20 mg of omeprazole.

**Figure 1:**
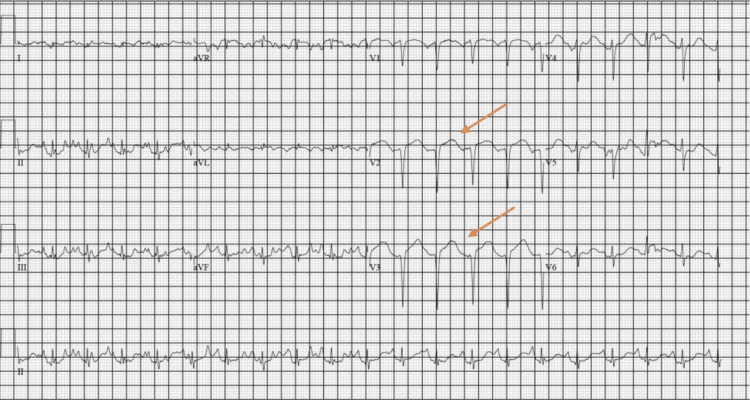
EKG on arrival with ST-segment elevations in V2 and V3

**Figure 2:**
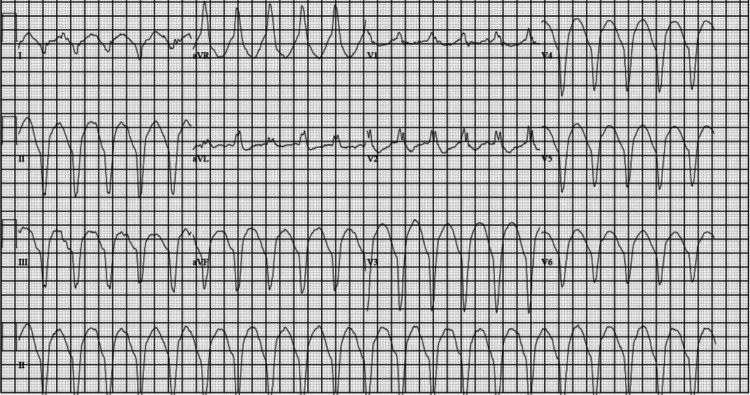
EKG after the return of spontaneous circulation (ROSC) showing wide complex tachycardia

**Figure 3:**
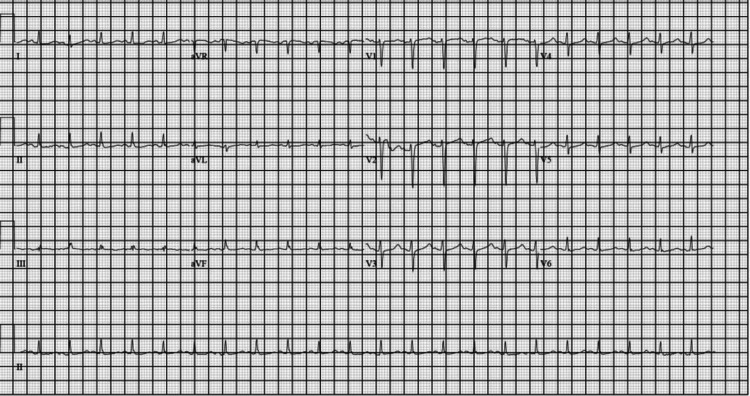
EKG after potassium replacement with wide complex tachycardia and ST elevations resolved

The patient’s triage vitals were as follows: blood pressure of 109/77 mmHg, heart rate of 122 beats per minute, temperature of 96.7°F, respiration of 36 breaths per minute, and oxygenation saturation of 100%. Given the patient’s neurological deficits and unclear time of onset, neurology was consulted and recommended head and neck CT angiograms. On arrival, the patient’s EKG showed corrected QT (QTc) prolongation, subtle ST depressions in the inferior leads, and J-point elevation in V2 and V3 (Figure 1), with undetectably high blood glucose. The troponin in the ED before cardiopulmonary resuscitation (CPR) was 40 ng/L (reference < 21 ng/L) and the urine toxicology screen came back negative. While at the CT scan, the patient was noted to go into a wide complex tachycardia. He was given 1 amp (50 mEq) of 8.4% sodium bicarbonate, 150 mg amiodarone, and calcium. The patient's rhythm had the appearance of TdP, then ventricular fibrillation (VF), and had no pulse. CPR was started; one epinephrine was given, and one shock was delivered. After resuming compressions, return of spontaneous circulation (ROSC) was achieved and the patient became alert and returned to the mental status he presented with. The patient’s rhythm continued to be a wide complex tachycardia consistent with VT (Figure 2). He was given 2 G magnesium as a concern for TdP, and an amiodarone drip was started. Synchronized cardioversion was attempted but the patient continued to stay in VT. The head CT was negative for acute stroke. Cardiology was consulted for concern on the initial EKG and VT/VF arrest. At that time, the patient’s venous blood gas results were potassium of 1.9 mmol/L (reference: 3.5-5.2 mmol/L). The patient appeared to have generalized seizure activity for approximately 30 seconds, which stopped prior to intervention. Potassium replacement was started, and the patient’s rhythm slowly began to narrow and converted to sinus tachycardia (Figure 3). Cardiology stated the likely etiology for the patient’s rhythm and arrest was hypokalemia and did not recommend cardiac catheterization. The patient had decreased mentation and the decision was made to intubate to protect the airway and predict the clinical course. A central line was placed, and the patient was persistently hypotensive after 3L resuscitation. The patient’s labs were consistent with hyperosmolar hyperglycemic nonketotic coma (HHNK). He was then admitted to the medical intensive care unit.

The patient was started on vasopressor support for continued hypotension after fluid resuscitation. He was found to have a splenic laceration on CT scans, and hemoglobin was monitored for a significant decrease or change in the serial abdominal exam. By the next day, his potassium was corrected, and the patient was started on insulin for HHNK treatment. The patient was also started on a sodium bicarbonate drip for persistent metabolic acidosis. He did not have any reoccurrence of VT after potassium replacement, and although the QT remained prolonged, the J point elevation that was noted on initial EKGs resolved on subsequent EKGs, and the cardiac workup (echocardiogram and troponin) did not reveal any other cause for rhythm and EKG abnormalities. The patient’s formal echocardiogram (transesophageal echocardiogram) did not have any wall motion abnormalities and had a normal ejection fraction. Specifically, the left ventricle was normal in size, and there was mild concentric hypertrophy. The systolic function was normal with an ejection fraction of 60-65%. The wall motion, systolic and diastolic functions, and right ventricle cavity were all normal.

After two days, the patient was noted to be febrile to 109°F and required increasing vasopressor support. He was ultimately placed on five vasopressors and stress dose steroids. The patient had worsening acute renal failure and worsening metabolic acidosis. The decision to pursue continuous renal replacement therapy (CRRT) was made; however, due to worsening hypotension, CRRT was not started, as the patient would not tolerate it. The patient’s family ultimately decided to transition to comfort care where he subsequently expired.

## Discussion

The differential diagnosis of regular wide complex tachycardia includes monomorphic VT, supraventricular tachycardia with aberrancy, an accelerated idioventricular rhythm, ventricular cardiac pacing, and antidromic re-entry tachycardia [8]. The presenting rhythm for this patient was monomorphic VT. VT accounts for roughly 8% of presenting wide complex tachycardia, the most common etiology of which is underlying ischemic heart disease [8]. Additional etiologies may include illicit sympathomimetic drug ingestion such as cocaine and methamphetamine, toxicological ingestions such as digitalis toxicity, and infiltrative cardiomyopathies such as lupus, sarcoidosis, and hemochromatosis [8]. Electrolyte abnormalities such as hyperkalemia, or more rarely hypokalemia, are also known to trigger VT [5]. In our patient, the QTc, especially when considered after rate correction, was long and likely contributed to ventricular arrhythmia. His troponin level was normal and his urine toxicology screen was negative. However, he had two medications that could have contributed to prolonged QTc. Haloperidol can cause QTc prolongation [9], and recent studies have reported that proton pump inhibitors (PPIs) such as omeprazole may also cause QTc prolongation through inhibition of the hERG-K channel [10]. The unidentified pills, empty haloperidol bottles, and prescribed omeprazole may presumptively be associated with the patient’s prolonged QTc.

In adolescents and young adults, other considerations must be considered with an EKG illustrating VT. Documented cases of VT have occurred secondary to myocarditis, hypertrophic cardiomyopathy, congenital long-QT syndrome, right ventricular cardiomyopathy, and congenital coronary artery anomalies [8]. Cases of congenital sodium channel cardiac mutations such as Brugada syndrome, characterized by a right bundle branch block pattern and ST segment elevations in V1-V3, have also been reported to devolve into VT causing sudden cardiac death [11]. This case is a unique instance, and one of few reported cases of hypokalemia resulting in VT.

Insulin deficiency is exacerbated when an inciting event leads to peripheral tissue “starvation,” which starts a cycle of glucose mobilization without glucose uptake resulting in a dysregulated state of hyperglycemia. HHS is usually defined by a glucose level above 600 mg/dL without ketoacidosis [7]. Diabetic ketoacidosis is defined by absolute insulin deficiency leading to hepatic ketone production because of peripheral lipolysis, with serum glucose above 250 mg/dL, high anion-gap metabolic acidosis, and ketonuria/ketonemia [12]. Intrinsic homeostatic adaptations lead to polyuria and polydipsia leading to severe dehydration and resultant weakness, malaise, and lethargy [7]. Osmotic diuresis leads to the significant elimination of body electrolyte stores (especially potassium) [7]. The neuropsychiatric presentation of HHS includes encephalopathy, seizures, hemichorea-hemiballismus syndrome, homonymous hemianopia, and coma [13]. Interestingly, several case studies have also found that HHS may present with reversible hemiparesis as a stroke mimic. It is currently postulated that hyperosmolality and hyper-viscosity lead to hypoxic-ischemic injury leading to reduced perfusion [14].

Previously reported cases involved hypokalemia in the setting of thyroid toxicity [15] and herbal toxicity [16], flecainide toxicity [17], and hyperemesis [18]. There are also examples of bidirectional VT and hypokalemia [19], as well as refractory TdP [20]. There are several documented examples of hypokalemia being associated with arrhythmia; in this case, the patient was thought to be profoundly hypokalemic due to HHNK. Our patient was also found to have neurologic deficits in the setting of hyperglycemia. There are also documented cases of craniofacial abnormalities in the setting of hyperosmolar hyperglycemia or just hyperglycemia, including hemifacial spasm [21]. The hyperosmolar hyperglycemic nonketotic syndrome causes hemichorea-hemiballismus [22], which is continuous, irregular, and involuntary jerking movements on one side of the patient’s body. Our patient was only noted to have twitching on one side of his face and the other with facial droop, which may have been more consistent with hemifacial spasm. This case was unusually complicated with HHNK and profound hypokalemia, emphasizing the fact that there is not always one causal etiology for the diagnostic challenges in critical patients. Hypokalemia is commonly encountered in clinical practice, but the clinical presentation of our case is rare.

## Conclusions

This case serves as an example of hypokalemia contributing to VT/VF/arrest and hypokalemia needs to be considered as a possible cause of arrhythmias. This case also serves as an example of neurological deficits in the setting of hyperosmolar and hyperglycemic setting, which needs to be considered as the possible cause when creating a differential in the presence of metabolic abnormalities and are different from the classic hyperglycemic hyperosmolar coma.

## References

[1] Castro D, Sharma S (2022). Hypokalemia. https://pubmed.ncbi.nlm.nih.gov/29494072/.

[2] Viera AJ, Wouk N (2015). Potassium disorders: hypokalemia and hyperkalemia. Am Fam Physician.

[3] Surawicz B (1974). Electrolytes and the electrocardiograim. Postgrad Med.

[4] Weiss JN, Qu Z, Shivkumar K (2017). Electrophysiology of hypokalemia and hyperkalemia. Circ Arrhythm Electrophysiol.

[5] Diercks DB, Shumaik GM, Harrigan RA, Brady WJ, Chan TC (2004). Electrocardiographic manifestations: electrolyte abnormalities. J Emerg Med.

[6] Skogestad J, Aronsen JM (2018). Hypokalemia-induced arrhythmias and heart failure: new insights and implications for therapy. Front Physiol.

[7] Adeyinka A, Kondamudi NP (2022). Hyperosmolar Hyperglycemic Syndrome. https://pubmed.ncbi.nlm.nih.gov/29489232/.

[8] Garner JB, Miller JM (2013). Wide complex tachycardia - ventricular tachycardia or not ventricular tachycardia, that remains the question. Arrhythm Electrophysiol Rev.

[9] Rahman S, Marwaha R (2022). Haloperidol. Haloperidol.

[10] Lazzerini PE, Cartocci A, Qu YS (2021). Proton pump inhibitors directly block hERG-potassium channel and independently increase the risk of QTc prolongation in a large cohort of US veterans. Circ Arrhythm Electrophysiol.

[11] El Sayed M, Goyal A, Callahan AL (2022). Brugada Syndrome. StatPearls.

[12] Lizzo JM, Goyal A, Gupta V (2022). Adult Diabetic Ketoacidosis. https://pubmed.ncbi.nlm.nih.gov/32809558/.

[13] Misra UK, Kalita J, Bhoi SK, Dubey D (2017). Spectrum of hyperosmolar hyperglycaemic state in neurology practice. Indian J Med Res.

[14] Marren SM, Beale A, Yiin GS (2022). Hyperosmolar hyperglycaemic state as a stroke cause or stroke mimic: an illustrative case and review of literature. Clin Med (Lond).

[15] Ghalyoun BA, Khaddash I, Shamoon D, Shaaban H, Hanna M, Tiyyagura S, Ismail M (2019). A rare case of hypokalemic ventricular tachycardia in a patient with thyrotoxic periodic paralysis. Int J Crit Illn Inj Sci.

[16] Akçay M, Yüksel S (2020). Resuscitated sudden cardiac death due to severe hypokalemia caused by teff grain herbal tea: a case report. Turk Kardiyol Dern Ars.

[17] Newson JM, Santos CD, Walters BL, Todd BR (2020). The case of flecainide toxicity: what to look for and how to treat. J Emerg Med.

[18] Kochhar PK, Ghosh P (2018). Ventricular tachycardia in a primigravida with hyperemesis gravidarum. J Obstet Gynaecol Res.

[19] Kulahcioglu S, Baysal PK, Kup A, Imanov E, Uslu A, Demir S, Gulsen K (2021). Bidirectional ventricular tachycardia induced by respiratory alkalosis mediated hypokalemia in a patient with acute ischemic heart failure. Pacing Clin Electrophysiol.

[20] Narang A, Ozcan C (2019). Severe Torsades de Pointes with acquired QT prolongation. Eur Heart J Acute Cardiovasc Care.

[21] Chen Y, Jin L, Xu Y, Li Z, Zhang B, Gao F (2021). Hyperglycemic hemifacial spasm: a case report. CNS Neurosci Ther.

[22] Balasubramaniyam N, Palaniswamy C, Rajamani VK, Subbiah G, Nivas J, Selvaraj DR (2011). Hyperosmolar hyperglycemic nonketotic syndrome presenting with hemichorea-hemiballismus: a case report. J Neuropsychiatry Clin Neurosci.

